# COVID-19 experience of people with severe mental health conditions and families in South Africa

**DOI:** 10.4102/sajpsychiatry.v30i0.2207

**Published:** 2024-04-09

**Authors:** Carrie Brooke-Sumner, Bongwekazi Rapiya, Bronwyn Myers, Inge Petersen, Charlotte Hanlon, Julie Repper, Laura Asher

**Affiliations:** 1 Mental Health, Alcohol, Substance Use and Tobacco Research Unit, South African Medical Research Council, Cape Town, South Africa; 2 Curtin Enable Institute, Curtin University, Perth, Australia; 3 Department of Psychiatry and Mental Health, Faculty of Health Sciences, University of Cape Town, Cape Town, South Africa; 4 Centre for Rural Health, College of Health Sciences, University of KwaZulu-Natal, Durban, South Africa; 5 Centre for Global Mental Health, Department of Health Service and Population Research, Institute of Psychiatry, Psychology and Neuroscience, King’s College London, London, United Kingdom; 6 Department of Psychiatry, College of Health Sciences, School of Medicine, Addis Ababa University, Addis Ababa, Ethiopia; 7 West London and St George’s Mental Health NHS Trust, London, United Kingdom; 8 Implementing Recovery Through Organisational Change (IMROC), Nottingham, United Kingdom; 9 Academic Unit of Lifespan and Population Health, School of Medicine, University of Nottingham, Nottingham, United Kingdom

**Keywords:** COVID-19, family caregivers, lived experience, lockdown, schizophrenia, South Africa, severe mental health condition, psychosis

## Abstract

**Background:**

People with severe mental health conditions, such as schizophrenia, and their family caregivers are underserved in low- and middle-income countries where structured psychosocial support in the community is often lacking. This can present challenges to recovery and for coping with additional strains, such as a pandemic.

**Aim:**

This study explored the experiences and coping strategies of people with lived experience of a severe mental health condition, and family caregivers, in South Africa during the initial stages of the coronavirus disease 2019 (COVID-19) pandemic.

**Setting:**

This qualitative study was conducted in the Nelson Mandela Bay District, Eastern Cape, South Africa, in the most restrictive period of the COVID-19 lockdown.

**Methods:**

Telephonic qualitative interviews were conducted with people with lived experience (*n* = 14) and caregivers (*n* = 15). Audio recordings were transcribed and translated to English from isiXhosa. Thematic analysis was conducted with NVivo 12.

**Results:**

Participants described negative impacts including increased material hardship, intensified social isolation and heightened anxiety, particularly among caregivers who had multiple caregiving responsibilities. Coping strategies included finding ways to not only get support from others but also give support, engaging in productive activities and taking care of physical health. The main limitation was inclusion only of people with access to a telephone.

**Conclusion:**

Support needs for people with severe mental health conditions and their families should include opportunities for social interaction and sharing coping strategies as well as bolstering financial security.

**Contribution:**

These findings indicate that current support for this vulnerable group is inadequate, and resource allocation for implementation of additional community-based, recovery-focused services for families must be prioritised.

## Introduction

People with severe mental health conditions, such as schizophrenia, may experience psychotic symptoms and cognitive impairment that can negatively impact social and role functioning.^1^ Personal recovery has evolved as a concept whereby people with a severe mental health condition engage in a lifelong process or journey of learning to live with the condition and experience meaning and fulfilment in life.^2^ In the early stages of the coronavirus disease 2019 (COVID-19) pandemic, the World Health Organization and research community called for urgent action to support vulnerable groups.^3^ This included support for people with severe mental health conditions in their recovery. There were concerns that the COVID-19 pandemic would affect population mental health determinants^4^ through exacerbating existing inequalities,^5^ restricting activities for sustaining livelihoods, and increasing unemployment, poverty and food insecurity. It was anticipated that the pandemic could accentuate the preexisting burden of elevated mortality,^6,7^ and structural exclusion from healthcare and development efforts^8,9^ among this group. In some settings, COVID-19 responses may have redirected scarce resources away from already inadequate mental health services, exacerbating barriers in accessing health and social care for people with mental health conditions.^5^ This may have increased the risk of relapse, for example, due to inadequate symptom management. Further, family caregivers in low- and middle-income countries (LMICs) often provide financial, practical, informational and social support, as well as creating structure for social relationships crucial for recovery.^10^ Caregivers already experienced stress in relation to these roles^11^ which may have been amplified by the impact of the pandemic. Challenging living circumstances in informal settlements, in which most of the population in LMICs live, may also have increased stress levels and reduced opportunities for social support.^12^ At the time of this study, lockdown restrictions in South Africa were in place which included closure of schools, businesses and retail outlets; prohibition of all public gatherings; ban on sale of alcohol and cigarettes; a curfew and requirement to stay at home except for visits to essential services (groceries, medications).^13,14^

Globally, there is now evidence that the COVID-19 pandemic has led to an increase in mental health conditions, particularly depression and anxiety. There is also evidence that people with severe mental health conditions are more likely to suffer from severe COVID-19 illness and death than the general population.^15,16,17^ This aligns with higher rates of comorbid conditions in this group, including cardiovascular diseases, type 2 diabetes and respiratory tract diseases.^18,19,20^ The onset of the COVID-19 pandemic also came after more than a decade of advocacy for the South African mental health budget (which has been historically allocated, in the main, to psychiatric hospital beds) to be more equitably balanced with a ring-fenced budget for community-based services. There continues to be a high rate (up to 30%) of service users in the country being repeatedly discharged into the community and subsequently rehospitalised (the ‘revolving door’ phenomenon)^21,22^ linked to inadequate support for personal recovery in the community.

This points to the need for evidence to inform programmes to support personal recovery in South Africa. There is however limited understanding of the lived experience of people with severe mental health conditions before, and since, the COVID-19 pandemic particularly in LMICs, including South Africa. This study was nested within the PRIZE study (peer-led recovery groups for people with psychosis in South Africa).^23^ PRIZE aimed to develop recovery support groups that would include people with lived experience of a severe mental health condition and their family caregivers together as group participants. To inform the PRIZE recovery groups, this study aimed to explore experiences and coping strategies of people with lived experience of a severe mental health condition, and family caregivers, in South Africa during the initial stages of the COVID-19 pandemic. Findings from the current study have informed development of the PRIZE recovery programme and materials.

## Research methods and design

### Setting

This study was conducted in the Eastern Cape province, Nelson Mandela Bay Metropolitan District in July 2020. In the district, there are 41 primary health clinics, 1 provincial hospital and 1 tertiary psychiatric facility. Eleven of these clinics provide some level of mental health services, including free medication for people with mental health conditions delivered by psychiatric nurses.

### Sample

Of the 11 clinics in the district providing mental health services, 8 had been previously identified as serving a predominantly isiXhosa-speaking population (the target population for the PRIZE study). Participants for the current study were drawn from the service users and caregivers who had previously participated in qualitative interviews for the PRIZE study, conducted by the PRIZE Project Coordinator (BR) and a Research Assistant. The original inclusion criteria were: (1) over the age of 18 years, (2) isiXhosa-speaking and (3) having a clinical diagnosis of schizophrenia or being a caregiver of a person with schizophrenia. The only exclusion criterion was not having capacity to provide informed consent. Using the contact records from the PRIZE study, a convenience sample was selected of participants who met the additional criteria: (4) having access to their own or a shared cell phone and (5) for service users, not being acutely unwell. Potential participants were contacted by the Research Assistant and invited to participate in a separate telephonic interview. The study was developed and is presented in line with COREQ guidance for reporting qualitative research.^24^

### Data collection

This study used an exploratory qualitative design. Qualitative interviews were conducted by BR (Project Coordinator) and the PRIZE Research Assistant. Both are experienced qualitative researchers with Honours degrees in Social Sciences, isiXhosa first language speakers, female, and both received training on the interview guide by C.B-S. Verbal telephonic consent to participate was obtained after an explanation of the study. Written informed consent was later obtained when participant time compensation vouchers (to the value of R150.00, approximately $8.00) were dispensed in person. The previous contact between researchers and participants helped to build rapport despite the telephonic format. Interviews were conducted in isiXhosa while participants were in a private space at their homes and lasted between 30 min and 40 min. Interviews followed a topic guide covering knowledge and concerns around COVID-19, experiences during lockdown, coping and resilience (see Online Appendix 1). Interviews were audio-recorded, transcribed and translated into English by BR and the Research Assistant, contributing to the initial familiarisation process.

### Analysis

Anonymised transcripts were stored and thematically analysed in NVivo 12 on password-protected laptops accessible only to the research team. C.B-S. and B.R. read all manuscripts for familiarisation and then independently coded the first five transcripts noting emerging themes. They then met to refine codes, with discussion focusing on codes with lowest level of agreement.^25,26^ Coding disagreements were resolved through consultation with LA. A final thematic framework was then agreed. Coding continued independently until saturation.

### Ethical considerations

Ethical approval for this study was granted by the research ethics committee of the South African Medical Research Council (EC007-4/2019/062020), and approval was granted by the Eastern Cape provincial and district health services.

## Results

Fourteen people with a severe mental health condition (service users) and 15 caregivers were interviewed (*n* = 29) out of 30 invited to participate. One service user declined indicating they were not interested in the study. Sociodemographic characteristics are listed in Table 1. Service user and caregiver participants expressed a range of experiences affecting their mental, physical and social well-being, as well as coping strategies in four emergent themes: (1) negative impact of the COVID-19 pandemic and lockdown measures, (2) coping strategies, (3) access to supportive services and (4) positive experiences during lockdown (Table 2).

**TABLE 1:** Sociodemographic characteristics of service user and caregiver participants.

Sociodemographic	Service users (*n* = 14)	%	Caregivers (*n* = 15)	%
Male	11	79	4	27
Female	3	21	11	73
**Age range**				
25 years or younger	1	7	0	0
26–35 years	4	29	2	13
36–50 years	7	50	4	27
51 years or older	2	14	9	60
**Relationship status**				
Single	12	86	7	47
Has a partner	2	14	0	0
Divorced/widowed	0	0	8	53

**TABLE 2:** Emergent themes from qualitative analysiṣ.

Theme		Subtheme
1.	Negative impact of the COVID-19 pandemic and lockdown measures	1.1. Material hardship 1.2. Intensifying social isolation 1.3. Mental health impact on service users 1.4. Mental health impact on caregivers
2.	Coping strategies	2.1. Finding support from others 2.2. Looking after yourself
3.	Access to supportive services	-
4.	Positive experiences during lockdown	-

COVID-19, coronavirus disease 2019.

### Theme 1: Negative impact of the coronavirus disease 2019 pandemic and lockdown measures

#### Subtheme 1: Material hardship

The majority of service user and caregiver participants described material hardship caused by lockdown. Most reported being sustained (pre-COVID and during lockdown) by government grants (e.g. disability grant, elderly persons’ grant). Financial effects included difficulty repaying loans. A minority described the negative financial impact of buying alcohol and cigarettes on the black market. Some caregiver participants experienced job losses (ad-hoc daily ‘piece work’) or reduced working days (e.g. domestic workers). Food insecurity was an important stressor for caregivers who shouldered responsibility for providing sufficient food for their whole household. A subgroup of caregiver and service user participants voiced disappointment at being excluded from government COVID-19 relief grants and food parcels:

‘I would ask myself, what will I do with these children? As a result, I applied for the food parcel and I did not even receive the necessary assistance from the government … I don’t know how they select … because what is important is that you get this money to live on this earth where your life doesn’t even count.’ (Caregiver 0015, female, unemployed)

#### Subtheme 2: Intensifying social isolation

Most service users reported that restrictions did not affect them, as they were largely isolated previously:

‘I am always indoors most of the time, I would go out now and then but not a lot. I spend a lot of my time here at home … it is how I was before.’ (Service user 004, female, unemployed)

By contrast, the majority of caregivers felt that the lack of social contact, communication and opportunities for recreation left a void in their lives. This prevented them from accessing (and giving) social support that would ordinarily have been found in social contexts. Restrictions caused distress to caregivers through preventing families from conducting traditional ceremonies (e.g. for funerals, including those for COVID-19-related deaths):

‘It [*lockdown*] has affected me a lot, I don’t even go to church as churches are closed. I’m just roaming around the house. Some people I know and love have passed away, but I can’t even go to the funerals because I’m afraid that maybe I’ll catch it.’ (Caregiver 18, female, unemployed)

#### Subtheme 3: Mental health impact on service users

Two service user participants described hearing voices, worries about being mentioned in social media and unusual beliefs around the causes of COVID-19. Several described fear of losing their lives, but most also expressed fear of losing family members particularly those with an income. This fear was driven by knowledge of the virus being ‘deadly’ and ‘airborne’ (Service user 0034, male, unemployed). By contrast, a subgroup of service user participants reported they did not worry about COVID-19; they felt it would not affect them because they were doing what was necessary to prevent infection.

‘So, my worry has been death of people also who are close to me since it is said that with this illness people who are at risk are the ones with other illnesses. So, I was worried about my mom … I would say I am feeling lost because of how quickly it spreads … it makes me feel like there is no hope.’ (Service user 0013, male, unemployed)

#### Subtheme 4: Mental health impact on caregivers

Caregiver participants commonly described experiences of poor mental health. They highlighted the additional care responsibilities and reduced opportunities for rest due to families being confined to their homes. One caregiver described having sought care (medication and two therapy sessions) for existing depression that was exacerbated during lockdown. Caregivers described ‘living in fear’ and high levels of stress driven by: (1) media reports; (2) lack of opportunity for ‘normal’ grieving processes for relatives; (3) fears for elderly family members and those with chronic conditions; (4) fears for their own health and that family members and others who did not take precautions would affect them and (5) future job security:

‘It [*the COVID-19 pandemic*] has affected me … I had so much fear especially when people close to me were dying … in my family, some people died and I was a mess … not even seeing the person’s coffin due to COVID-19 regulations, it was traumatising. That process stressed us a lot as a family.’ (Caregiver 0027, female, unemployed)

In relation to their responsibility for caring for a family member with a mental illness, caregiver participants identified additional stress points including: (1) worries about how their family member would access their medication and (2) fears that they would be more vulnerable to COVID-19 infection because of not adhering to social distancing and mask-wearing guidance.

### Theme 2: Coping strategies

Both service user and caregiver participants conveyed adaptive coping strategies. These fell broadly into strategies to promote mental or physical well-being.

#### Subtheme 1: Finding support from others

The most commonly described coping strategy for service users and caregivers was receiving or seeking social support from others such as family members and neighbours. Checking up on each other was commonly reported and provided comfort and a sense of being cared for. Contact was made through text messages, phone calls and WhatsApp messages. A commonly described strategy by caregiver participants was keeping themselves occupied with productive activities such as household tasks, knitting, sewing, crochet and gardening. Both service users and caregivers found TV and radio and listening to music important sources of entertainment, although one caregiver reported making a conscious decision to stop watching TV or listening to radio news as a way to reduce anxiety. Some caregiver participants and a minority of service user participants emphasised religious faith as an anchor for coping. This was founded on the need to trust God in a situation beyond their control. Other caregivers and service users described developing acceptance of their lack of control by focusing on positivity, self-motivation and new ways of keeping healthy, not related to religious faith.

‘But after some time you have to come to the conclusion that you can stress as much as you want, it is how it is. So, in order for you to not feel the situation you have to accept how things are, you are not in control, only God is in control … there is no prediction, we are living the day as it is.’ (Caregiver 0026, female, unemployed)

#### Subtheme 2: Looking after yourself

Service user and caregiver participants recognised the benefit of building their physical health. All participants had knowledge of COVID-19 symptoms and transmission, with just a few misconceptions (e.g. that COVID-19 causes asthma). TV and radio were the principal sources of this information. Service user and caregiver participants translated the health education advice they received into a sense of empowerment that they were doing what they could to protect themselves (mask wearing, sanitizing, social distancing, staying home). Some disagreements between family members were described, relating to the degree of infection risk control with caregivers who were older family members being more cautious than young people. Use of immune-boosting herbal remedies was commonly reported (e.g. lemon, garlic, ginger) and *umhlonyane* (African wormwood). Some caregivers described weight gain in themselves and family members ‘because you are always sitting at home and there is nothing to do’ (Caregiver 0015, female, unemployed). One service user described how his concern over COVID-19 had encouraged him to consider restarting treatment for human immunodeficiency virus (HIV):

‘I have realised that I need to do things right. Maybe this treatment that I have not been using for HIV, I should start using it again so that I am not at risk of this virus. I have to go and get the treatment for HIV at the clinic, but I am afraid that the nurse is going to shout at me.’ (Service user 001, male, unemployed)

### Theme 3: Access to supportive services

On the one hand, most service user and caregiver participants described few challenges accessing grants. A minority of caregivers and service users, however, described being fearful of collecting grant payments as they wished to avoid long queues and were confused over collection dates. Several service users had never received grants and were unsure how to rectify this:

‘I don’t feel alright because I have given up on it [*getting the disability grant*]. There is another guy who stays close to my home, he is on the same treatment as I am. He says that it has been four years since he has stopped taking them [*medication*], but he says that he [*still*] gets a grant’. (Service user 0011, male, unemployed)

In relation to health services, some service user participants described being able to collect their medication without problems, with medication being packaged for 2 months’ supply. Caregivers also described being able to collect other chronic disease medications as usual (e.g. for diabetes, hypertension). However, many service users experienced problems accessing the clinics where they would usually collect medication. Where clinics were open, the service was limited to dispensing of medication with no time for consultation. Some described long waiting times and not being attended to on their first clinic visit (despite being given an appointment). These service changes had some tangible impacts on service users. One service user described having no medication for 3 weeks. One described concerns about symptoms and difficulty in managing medication side effects but being unable to see the doctor:

‘As early as 05:00 we are outside at the clinic. We are getting cold standing with the children and we would get attended to at 09:00 when they arrive then we would be allocated to our respective queues all to be returned home. They say that we cannot be seated the way we used to be seated.’ (Caregiver 0020, female, unemployed)

### Theme 4: Positive experiences during lockdown

Some caregiver and service user participants reported that they had no positive experiences, but also noted that they had not experienced an increase in family conflicts. Others related how enforced time together had positive effects on family relationships and the home environment, including sharing of household tasks with defined roles and responsibilities:

‘It [*lockdown*] has been good for us … we were really happy, we played cards and games … and we bonded very well.’ (Service user 008, male, unemployed)

A subgroup of both caregivers and service users emphasised that the alcohol ban and accompanying policing had a positive impact on their households and communities because of: (1) reduced noise levels, street violence and litter; (2) fewer alcohol-related arguments between family members and (3) individuals’ own reduced alcohol consumption. A minority of service user and caregiver participants described saving money due to reduced transport costs, lower food costs due to fewer visitors to the household, and reduced spending on alcohol and cigarettes. A minority of both service user and caregiver participants also described experiences of personal growth, which they related to being less busy and having more time to ‘talk and reflect on life’ (SU00 14, male, unemployed)

‘The positive is that I get to spend more time with my family. I also get the chance to analyse myself and the situation that I am involved in, you understand. So it has it’s good and bad.’ (Caregiver 007, female, unemployed)

## Discussion

This study explored how service users with severe mental health conditions and their caregivers living in an underserved area in South Africa experienced the initial stages of the COVID-19 lockdown. Our findings contribute to the limited evidence on pandemic-related adversities for this group. In the study, service users and caregivers commonly described increased material hardship and the related stress this brought. Caregiver participants described intensified social isolation and heightened anxiety, particularly in relation to having multiple caregiving responsibilities. Coping strategies were identified by both service users and caregivers and included finding ways to get support from others and also give support, engaging in productive activities and taking care of physical health.

Evidence from high-income countries (HICs) indicates that in some contexts, people with schizophrenia coped with the stress and anxiety experienced during the pandemic similarly to the general population.^27,28^ Research about the mental health impacts of the COVID-19 pandemic has largely neglected to examine the impacts on people with severe mental health conditions in LMIC who face preexisting psychosocial challenges and inequalities.^29,30^ People with severe mental health conditions and their families are already some of the most economically deprived.^31^ Similar to experiences in the United Kingdom, our findings suggest COVID-19 has increased this group’s risk of deepening poverty.^32^ However, our study highlights that the potential sequelae in LMIC are severe, particularly in terms of food security.

Apart from financial deprivation, service user participants described social isolation which may increase symptom severity.^33,34^ Although most service users felt their social isolation was already high and largely unchanged, this is concerning as social connection is an important part of recovery in both HICs and LMICs.^27,30,35,36^ This finding indicates an important gap in care in South Africa and suggests the current biomedical orientation fails to meet needs for social connection.^10,37^

Preliminary data from high-income settings suggested an increased risk of relapse during the initial stages of the COVID-19 pandemic.^38^ Only a minority of service users in this study described exacerbations of psychotic symptoms and unaddressed side effects, similar to HIC findings.^39^ Nonetheless, service user participants described fewer opportunities for consultation with health workers and changes in the clinic organisation that impacted on their access to ongoing mental health care. These barriers to care may have a continuing contribution to delayed health-seeking behaviour, with the potential to further entrench health inequalities in this vulnerable group.^40,41^

Caregiver participants described worsened mental health during this period. They described how their anxiety was intensified, partly related to their responsibility for providing for the basic needs of their households (not only their family member with a mental health condition). They also experienced diminished opportunities for accessing social support networks, similar to findings from other LMICs.^42^ Before COVID-19, caregivers were already vulnerable to stress, anxiety and depression.^43,44^ In LMICs such as South Africa, family caregivers have a pivotal role in taking care of people with severe mental health conditions. The pandemic may have had more pronounced impacts on caregivers in these settings compared to in HICs. In this study, distress caused by the inability to conduct traditional burial ceremonies was also strongly highlighted. The significance of burial rituals and shared grief in isiXhosa culture has been documented.^45^ Absence of these rituals and social processes contributed to the protracted impact of the pandemic and may have led to a reservoir of unresolved grief.

In common with studies from HIC,^18,28,46^ our findings suggest that some service user and caregiver participants demonstrated flexibility and resilience.^47^ Caregivers in particular described the role of religion in coping with feelings of lack of control and assisting them in finding acceptance of uncertainty. Religious faith can be a key coping strategy for people with mental health conditions and families in South Africa^48^ and some other LMICs.^49,50^ Its function of being a route to finding meaning and hope, as well as a source of support and social connection, can be a key aspect of recovery.^10,51,52^ Our findings indicate assimilation of public messaging relating to increased risk of worse outcomes for people with chronic conditions. South Africa was one of only a handful of nations globally that implemented an alcohol ban during the pandemic. Service user and caregiver participants reported positive experiences of the ban on alcohol sales (improved safety and increased disposable income) pointing to the far-reaching negative impact alcohol has on families and communities in South Africa,^53,54,55^ and the benefits of limiting alcohol use for promoting recovery and improvements in other health outcomes.

Several broad implications stem from this study which align with the call for context-appropriate mental health systems supporting the most vulnerable during future pandemics.^4^ The pressing concerns around material needs indicate the importance of providing for basic needs of the most vulnerable in society as a starting point for coping with a pandemic. Findings on the importance of spirituality point to pandemic responses that build on context-specific, culturally relevant anchors for coping, which will differ from country to country. Coping strategies highlighted through this study, and in line with phenomenological studies from HIC,^27,28^ need to be harnessed and supported as society realigns following COVID-19. Our findings show the need for strengthening mental health services for people with severe mental health conditions. Specifically, interventions that address unmet needs for social connection and that assist caregivers are required. Caregivers need support for their own mental health concerns and for their role as primary sources of support. Intersectoral collaboration between health, social development and nongovernmental sectors^3,56,57^ is needed to support implementation of these interventions and ensure adequate provision of community mental health services.

### Limitations

The timing of the study meant that only people who were contactable via cell phone were included. Some participants in this setting would have been inaccessible by cell phone. This may have introduced homogeneity in the social environments of participants and a bias towards those in families with more resources. There may have been a response bias due to interviewers being known to participants from previous research activities.

## Conclusion

We have presented experiences of people with severe mental health conditions and their caregivers during the COVID-19 lockdown in underserved settings in the Eastern Cape. The study has highlighted the adversity related to material hardship, needs for social support and connection, as well as potential for resilience and coping in the study participants. Several aspects of these experiences were similar to those reported in HIC. However, some negative impacts may have been more prominent in the South African context. The powerful mental health impacts of the pandemic on caregivers may reflect the already heavy burden of care experienced by this group. Material hardship and negative impacts on mental health for people with mental health conditions and their caregivers highlighted that support available to them remains inadequate. Implementation of community-based supportive services that respond to social needs alongside biomedical mental health care must be prioritised.
